# Broadband Circularly Polarized Antenna Array with Sequential Rotation Feeding and a Windmill-Shaped Defected Ground Structure

**DOI:** 10.3390/mi17060666

**Published:** 2026-05-28

**Authors:** Shiquan Zhang, Shuaijie Wu, Xianqiong Wen, Hongxing Zheng

**Affiliations:** 1 School of Intelligent Science and Engineering, Xi’an Peihua University, Xi’an 710125, China; wenxianqiong@163.com; 2 China Mobile Group Hebei Co., Ltd., Handan Branch, Handan 056000, China; wushuaijie_hd@he.chinamobile.com; 3 School of Electronic Information Engineering, Hebei University of Technology, Tianjin 300401, China

**Keywords:** array antenna, circular polarization, sequential rotation, defected ground structure, communication test, packet loss rate

## Abstract

To address the demanding requirements for high gain, wide bandwidth, and stable circularly polarized (CP) radiation in wireless local area network (WLAN) applications, this paper proposes and implements a broadband circularly polarized array antenna primarily targeting the 2.4–2.484 GHz ISM band. The design employs a coplanar waveguide fed broadband CP monopole antenna as the radiating element. A sequential rotation technique is utilized to form a four-element array, and a windmill-shaped defected ground structure is introduced to further extend the bandwidth. The antenna is fabricated on a low-cost FR4 substrate with overall dimensions of 0.98λ_0_ × 0.98λ_0_ × 0.008λ_0_ at 2.4 GHz. Simulation and measurement results show that the array antenna achieves a −10 dB impedance bandwidth of 1.22–2.78 GHz (87.1% relative bandwidth) and a 3-dB axial ratio bandwidth of 1.85–2.66 GHz (35.0% relative bandwidth), ensuring sufficient margin over the target WLAN band. At the center frequency of 2.45 GHz, the antenna exhibits left-hand circular polarization radiation, with a measured peak gain of 8.2 dBic and a cross-polarization discrimination better than 20 dB. To verify its performance advantages in practical systems, the designed antenna was integrated into a ZigBee wireless communication system for data transmission testing. Under controlled conditions, the system employing the proposed antenna achieves a packet loss rate of 3.0% ± 0.4% in a complex multipath environment, significantly outperforming a traditional linear-polarized whip antenna (19.0% ± 1.1%). The results demonstrate that the proposed antenna, featuring wide bandwidth, high gain, and strong anti-interference capability, is a robust solution for WLAN access points and internet of things gateways.

## 1. Introduction

With the rapid development of fifth-generation (5G) mobile communication and Internet of Things (IoT) technologies, WLANs, as crucial access and backhaul means, impose higher performance requirements on terminal antennas. In complex indoor and outdoor propagation environments, multipath effects and polarization mismatch can lead to severe signal attenuation and fading. The CP waves, capable of receiving incoming waves of arbitrary polarization and reversing their handedness upon reflection, can effectively suppress multipath interference, thereby significantly enhancing the stability and reliability of communication links [1,2]. However, a single antenna element often suffers from limited gain and narrow bandwidth, making it difficult to meet the demands of medium-to-long distance and high-rate communication. Array antennas, by arranging and exciting multiple radiating elements in a specific manner, can achieve beamforming, high gain, and stable radiation characteristics, offering an effective solution to the aforementioned problems [3,4].

Nevertheless, the design of broadband circularly polarized array antennas faces several challenges. Firstly, the inevitable mutual coupling between elements, especially when the element spacing is less than half a wavelength, can degrade the antenna’s impedance matching and circular polarization performance [5]. Secondly, the design of a feed network that provides equal amplitude and specific phase difference excitation to the array is complex, and its bandwidth limitation often becomes a bottleneck constraining the overall performance of the array [6]. Recent advances in array design have focused on bandwidth enhancement techniques. Sequential rotation (SR) technology, as an effective arraying method, can significantly broaden the axial ratio bandwidth of the array by physically rotating the array elements clockwise (or counterclockwise) and providing corresponding phase feeding, thereby compensating for the axial ratio fluctuations of the individual elements [7,8]. Additionally, introducing a defected ground structure (DGS) on the ground plane, by altering the ground current distribution, can excite additional resonant modes or improve impedance matching, which is a common method for extending antenna bandwidth [9]. More recent studies have explored combining these techniques. For instance, references [10,11] demonstrated the use of SR with different element types, while [12] investigated novel DGS geometries for wideband performance. Furthermore, system-level validation of CP antenna performance in real-world IoT scenarios, as highlighted in [13], remains an area of active research.

Based on the above analysis, this paper presents a broadband circularly polarized array antenna suitable for the 2.4 GHz WLAN band. While DGS-based bandwidth enhancement is well-established, the key contribution of this work lies in the specific integration of a windmill-shaped DGS with an SR-fed CP array. The key innovation of this work is the novel integration of a windmill-shaped DGS with the SR-fed array. This combination is optimized to perturb the ground current distribution synergistically with the SR excitation, achieving broader axial ratio (AR) bandwidth than previously reported configurations. Furthermore, system-level validation of CP antenna performance in real-world IoT scenarios remains an active research area [14]; thus, this paper details the design process and validates the antenna through comprehensive communication tests.

To further enhance performance, the ground plane is innovatively modified into a windmill-shaped DGS. Consequently, the antenna achieves a wide impedance bandwidth (87.1%) and a wide axial ratio bandwidth (35.0%) while obtaining a relatively high gain of 8.2 dBic. This paper details the design process of the antenna element, feed network, and the overall array, and verifies the effectiveness and superiority of the design through comprehensive parameter simulation analysis and physical testing. A significant contribution of this work is the practical system-level evaluation. To further assess the performance gain of the designed antenna in actual wireless communication systems, it was applied to a ZigBee wireless sensor network for comparative communication testing. The packet loss rate, a key metric, is used to quantitatively analyze the anti-interference advantage of circularly polarized antennas relative to traditional antennas in complex environments.

The remainder of this paper is organized as follows. Section 2 details the structural design of the circularly polarized antenna element, the sequential rotation feed network, and the implementation of the windmill-shaped DGS. Section 3 presents a parametric study analyzing the influence of key geometric dimensions on antenna performance. Section 4 discusses the fabrication of the prototype and compares the measured results with simulations, including impedance, axial ratio, and radiation patterns. Section 5 establishes a ZigBee-based wireless communication platform to evaluate the packet loss performance of the proposed antenna against reference antennas in both open and complex multipath environments. Finally, Section 7 concludes the paper by summarizing the achievements and potential applications of this work.

## 2. Antenna Element and Array Design

### 2.1. Design of the Broadband Circularly Polarized Antenna Element

**Figure 1:**
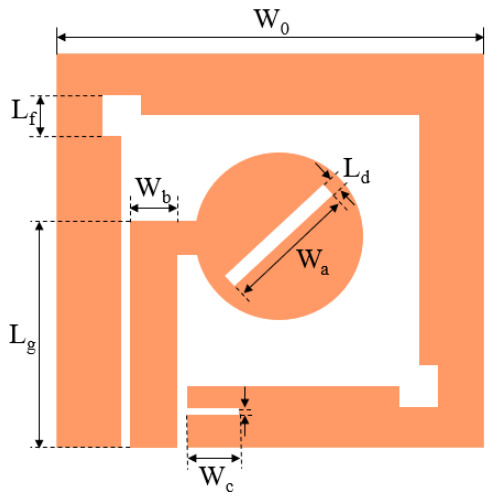
Geometry and configuration of the proposed CPW-fed broadband circularly polarized antenna element.

The radiating element is the fundamental building block determining overall array performance. The structure of the element antenna designed in this paper is shown in Figure 1. The antenna uses a low-cost FR4 substrate with a dielectric constant ε_r_ = 4.4 and thickness h = 1 mm. The radiator is a circular patch loaded with a rectangular slot, fed by a coplanar waveguide (CPW). This feeding scheme is chosen for its advantages such as design flexibility, easy integration, and good dispersion characteristics, which help achieve broadband performance [15]. The antenna’s ground plane extends upwards, partially surrounding the radiating patch to form a monopole structure. To achieve circularly polarized radiation and expand bandwidth, three key design features are implemented: (1) The CPW feedline is laterally offset to achieve asymmetric feeding, exciting two orthogonal degenerate modes; (2) A pair of square slots are loaded symmetrically on the ground plane near the radiating patch to optimize CP purity; (3) A rectangular opening slot is loaded at the bottom of the ground plane to further perturb the surface current distribution, extending both impedance and axial ratio bandwidth. After optimization using electromagnetic simulation software, the key dimensions of the antenna element are listed in Table 1.

**Figure 2:**
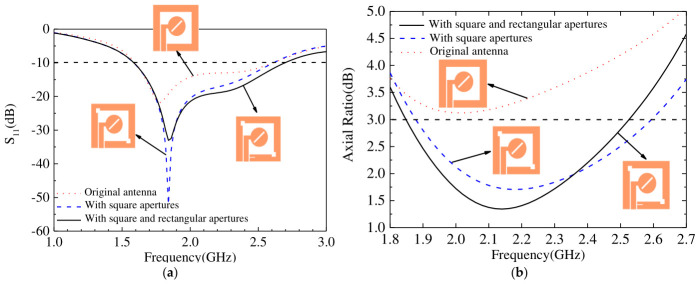
Evolution of the antenna element structure, I (Basic), II (with square slots), III (Final with all slots). (**a**) Simulated reflection coefficient (S_11_) and (**b**) axial ratio (AR) for the three structural evolution stages.

To clarify the design rationale, Figure 2a,b illustrate the evolution process of the antenna element and its impact on S_11_ and axial ratio. It can be seen that the original structure (without any slots) has poor impedance matching, and its axial ratio is far above 3 dB. Loading a pair of symmetric square slots significantly reduces the axial ratio near 2.2 GHz, improving the CP characteristics. Adding the bottom rectangular slot on this basis further reduces S_11_ across the entire frequency band and effectively expands the axial ratio bandwidth, verifying the performance enhancement effect of the DGS.

**Figure 3:**
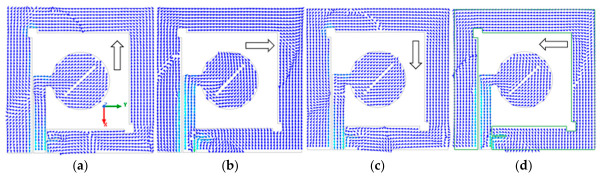
Simulated surface current distribution on the radiating element at 2.2 GHz for phase angles of (**a**) 0°, (**b**) 90°, (**c**) 180°, and (**d**) 270°, the direction of the surface current of the antenna demonstrating left-hand circular polarization (LHCP).

Figure 3 shows the surface current distribution of the element at the 2.2 GHz frequency point at four cases of instants: 0°, 90°, 180°, and 270°. The current vector rotates clockwise along the +z-axis, conforming to the left-hand rule, indicating that this element radiates left-hand circularly polarized waves.

### 2.2. Sequential Rotation Array and Feed Network Design

**Figure 4:**
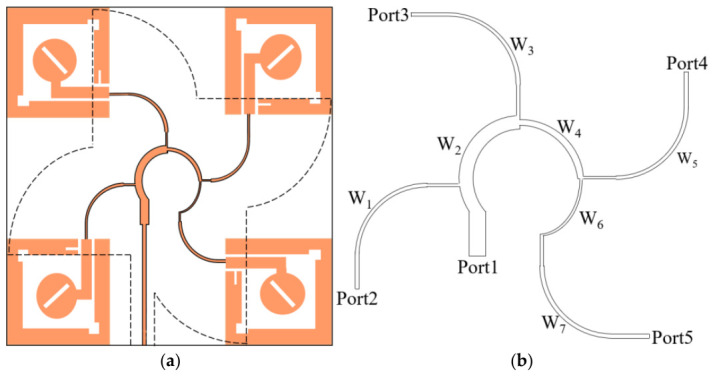
Configuration of the proposed 2 × 2 array antenna, (**a**) Top view layout showing the sequential rotation of the four elements, (**b**) Detailed geometry of the series microstrip line feed network designed for equal power division and 90° sequential phase shift with seven quarter-wavelength impedance transformers.

The sequential rotation technique is employed to array four of the aforementioned element antennas. The array configuration is shown in Figure 4a. The four elements are placed at the corners of a 126 mm × 126 mm FR4 substrate with an inter-element spacing of 45.6 mm (approximately 0.33λ_0_, where λ_0_ is the free-space wavelength at 2.2 GHz). Each element is sequentially rotated clockwise by 0°, 90°, 180°, and 270°. The SR technique itself inherently improves the axial ratio bandwidth of the array [16]. To provide the four elements with equal amplitude and sequentially lagging 90° phase excitation, a series microstrip line feed network is designed, as shown in Figure 4b. This network consists of seven quarter-wavelength impedance transformers with different characteristic impedances, connected by curved microstrip lines to minimize parasitic effects caused by discontinuities. Among them, three main transmission lines with gradually varying widths (W_2_, W_4_, W_6_) generate the required progressive phase delay, while four parallel branches (W_1_, W_3_, W_5_, W_7_) are responsible for impedance transformation, ultimately achieving equal power division and the 90° stepped phase difference at the four output ports. The principle of network is analyzed as follows.

**Figure 5:**
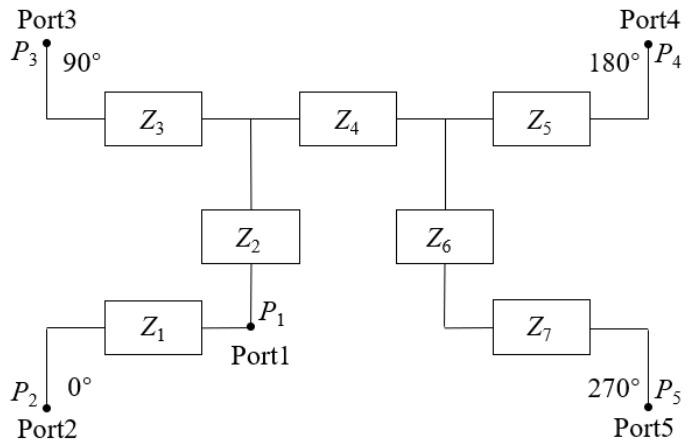
The working principle of the feed network, quarter-wavelength impedance transformer of the characteristic impedances of each microstrip line.

The working principle of the feed network is shown in Figure 5. It illustrates the operating principle. Since the power is equally divided at ports 2–5, we have

(1) $$P_{2}=P_{3}=P_{4}=P_{5}=P_{1}/4$$

Assuming the input impedances of transmission lines W_2_, W_4_, and W_6_ are Z_in2_, Z_in4_, and Z_in6_, and their input powers are P_in2_, P_in4_, and P_in6_, respectively. The input impedances of each parallel stub are Z_in1_, Z_in3_, Z_in5_, and Z_in7_, with their corresponding input powers being P_in1_, P_in3_, P_in5_, and P_in7_. Then we have

(2) $$P_{in6}=P_{5},P_{in4}=P_{in6}+P_{4},P_{in2}=P_{in4}+P_{3},P_{1}=P_{in2}+P_{2}$$

**Figure 6:**
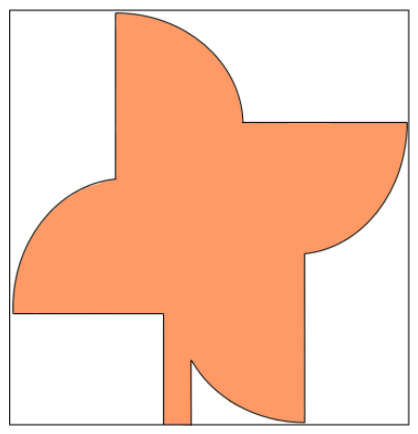
Bottom view showing the innovative windmill-shaped defected ground structure (DGS).

Based on the circuit analysis shown in Figure 6, we can calculate

(3) $$Z_{in1}=3Z_{in2},Z_{in3}=2Z_{in4},Z_{in5}=Z_{in6}=Z_{in7}$$

Assuming Z_in_, Z_0_, and Z_l_ represent the microstrip line input impedance (edge impedance), characteristic impedance (50 Ω), and the characteristic impedance of the quarter-wavelength impedance transformer, respectively, with *l* being the microstrip line length and *β* the phase constant of the transmission line, the impedance matching calculation formula for the microstrip line is

(4) $$Z_{in}=Z_{o}\frac{Z_{L}+jZ_{o}\tan(\beta l)}{Z_{o}+jZ_{L}\tan(\beta l)}$$

For the quarter-wavelength impedance transformer, i.e., when the transmission line length l is a quarter-wavelength, Equation (4) can be written as

(5) $$Z_{L}=\sqrt{Z_{o}Z_{in}}$$

Therefore, based on the above formulas, after calculation and optimization, the characteristic impedances of each microstrip line section are Z_1_ = 101.5 Ω, Z_2_ = 78.3 Ω, Z_3_ = 131 Ω, Z_4_ = 97.5 Ω, Z_5_ = Z_7_ = 80.4 Ω, and Z_6_ = 119 Ω.

The sequential rotation technique is employed to array four of the aforementioned element antennas. To provide the four elements with equal amplitude and sequentially lagging 90° phase excitation, a series microstrip line feed network is designed.

To address the inherent narrowband nature of SR feed networks and validate the broadband performance claim (87.1% impedance bandwidth), the simulated amplitude and phase responses of the four output ports were analyzed across the operating band (Figure 5). The results demonstrate that the amplitude imbalance remains within ±0.5 dB and the phase deviation stays within ±10° from 1.8 GHz to 2.6 GHz. This confirms that the SR technique maintains effective quadrature excitation across the majority of the impedance bandwidth, ensuring stable CP radiation even at the band edges (e.g., 1.5 GHz and 2.7 GHz).

### 2.3. Windmill-Shaped Defected Ground Structure

To further enhance the bandwidth of the array antenna, the overall ground plane is innovatively modified. The traditional solid rectangular ground plane is etched into a windmill-shaped structure, as shown in Figure 6.

This windmill-shaped DGS is equivalent to introducing multiple resonant units, which perturb the original distribution path of the ground current, excite new resonant modes, and couple with the antenna element itself and the sequential rotation structure, thereby synergistically expanding the overall impedance and axial ratio bandwidth [17].

**Figure 7:**
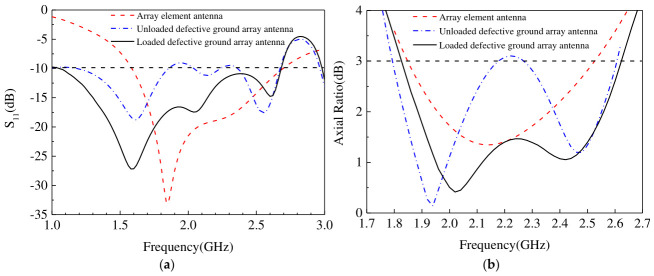
Comparison of simulated performance: (**a**) Reflection coefficient (S_11_) and (**b**) Axial ratio (AR) for a single element, the 2 × 2 array with a solid ground plane, and the proposed 2 × 2 array with the windmill-shaped DGS.

Figure 7 compares the simulated S_11_ and axial ratio performance of (i) a single array element, (ii) the 2 × 2 SR array with a conventional solid ground, and (iii) the proposed 2 × 2 SR array with the windmill-shaped DGS. The results demonstrate that after loading the DGS, the −10 dB impedance bandwidth of the array increases significantly, and the 3-dB axial ratio bandwidth is substantially expanded, fully proving the effectiveness of this integrated design approach.

**Figure 8:**
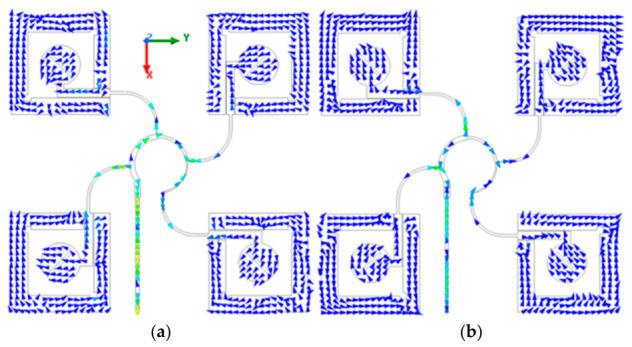
Surface current diagrams of the array antenna at different phases at 2.2 GHz, (**a**) Current flow direction on the antenna surface at 0°, (**b**) Current flow direction on the antenna surface at 90°.

Figure 8 displays the surface current distribution of the array antenna at 2.2 GHz for different phases. The figure shows that the current directions on the antenna surface are orthogonal to each other at 0° and 90°, and they rotate clockwise around the +z direction. Consequently, the antenna satisfies the left-hand rule in the +z direction, thus exhibiting left-hand circular polarization.

## 3. Simulation Analysis and Parameter Study

**Figure 9:**
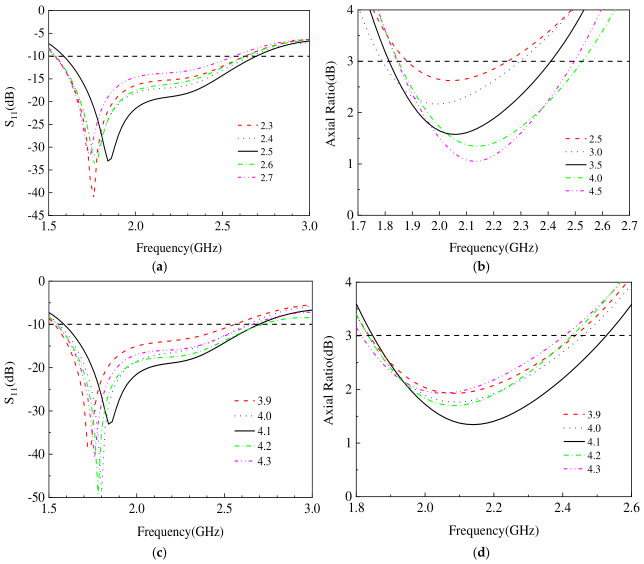
Parametric study of the antenna element. Simulated (**a**) reflection coefficient (S_11_) and (**b**) axial ratio (AR) for different lengths (L_f_) of the square slot on the ground plane. Simulated (**c**) S_11_ and (**d**) AR for different widths (Wc, unit: mm) of the rectangular slot at the bottom of the ground plane.

A three-dimensional electromagnetic simulation software was used to model and perform full-wave simulation of the array antenna. The effects of two key parameters—the length of the square slot on the ground plane (L_f_) and the width of the rectangular slot (Wc)—on the antenna performance were studied, as shown in Figure 9.

Figure 9a shows that as the square slot length L_f_ increases from 2.3 mm to 2.7 mm, the antenna’s resonant frequency shifts to lower frequencies, and Figure 9a shows the axial ratio near 2.2 GHz first decreases and then increases. When L_f_ = 2.5 mm, the antenna obtains the best impedance matching and the lowest axial ratio within the target frequency band. Figure 9c shows that the rectangular slot width Wc mainly affects the matching at higher frequencies. As Wc increases from 3.9 mm to 4.3 mm, the high-frequency resonance points shift, and the Figure 9d shows axial ratio bandwidth also changes accordingly. Choosing Wc = 4.1 mm provides good comprehensive performance across a wide frequency band. This analysis provides the basis for determining the final antenna dimensions.

## 4. Measurement Results and Discussion

**Figure 10:**
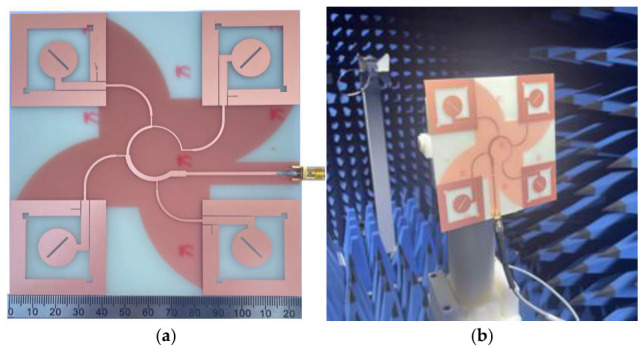
Fabrication and measurement setup, (**a**) Photograph of the fabricated antenna prototype and (**b**) the anechoic chamber test environment.

To verify the correctness of the simulation design, the antenna was fabricated and its performance was tested in an anechoic chamber. The antenna prototype and test environment are shown in Figure 10.

### 4.1. Impedance and Axial Ratio Characteristics

**Figure 11:**
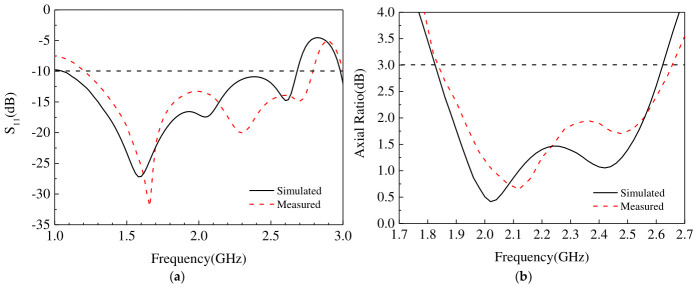
Simulated and measured reflection coefficient (S_11_) and axial ratio (AR) of the proposed array antenna, (**a**) S_11_ and (**b**) axial ratio.

A vector network analyzer was used to measure the return loss S_11_ of the antenna. The results are shown in Figure 11a. The measured −10 dB impedance bandwidth is 1.22–2.78 GHz, which agrees well with the trend of the simulation results (1.05–2.67 GHz) and completely covers the 2.4–2.484 GHz WLAN band. The measured bandwidth is slightly wider than the simulation, which may be due to minor deviations between the actual substrate parameters and the simulation settings, as well as parasitic effects introduced by soldering the SMA connector. The measured axial ratio results are shown in Figure 11b. The measured 3-dB axial ratio bandwidth is 1.85–2.66 GHz, highly consistent with the simulated prediction of 1.84–2.62 GHz, demonstrating the effectiveness of the sequential rotation technique and the DGS in broadening the axial ratio bandwidth.

### 4.2. Radiation Pattern and Gain

**Figure 12:**
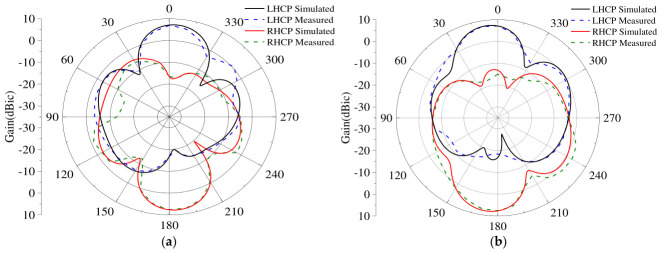
Measured radiation patterns of the proposed antenna at 2.2 GHz: (**a**) E-plane (xoz plane) and (**b**) H-plane (yoz plane) patterns, showing co-polarization (LHCP) and cross-polarization (RHCP) components.

**Figure 13:**
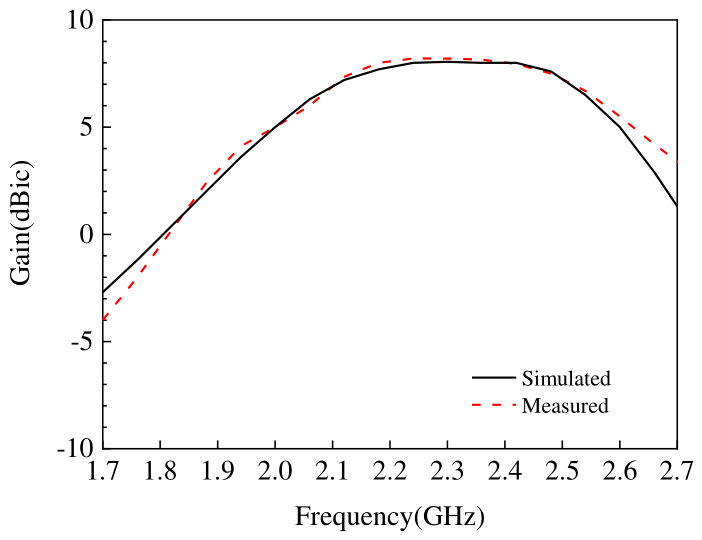
Simulated and measured peak gain of the proposed broadband circularly polarized array antenna across the operating frequency band.

The radiation patterns of the antenna at the center frequency of 2.2 GHz were measured in the anechoic chamber, as shown in Figure 12. The antenna exhibits good directional radiation characteristics in both the E-plane and H-plane, with high front-to-back ratio and symmetrical patterns. In the main radiation direction (+z direction), the left-hand circularly polarized (LHCP) component is significantly higher than the right-hand circularly polarized (RHCP) component, with a cross-polarization isolation better than 15 dB, indicating high purity of the radiated CP wave. The simulated and measured gain of the antenna is compared in Figure 13. Within the operating band, the gain curve is relatively flat, with a measured peak gain of approximately 8.2 dBic, basically consistent with the simulation results, meeting the gain requirements for WLAN access points.

## 5. Antenna Prototype and Communication System Testing

**Figure 14:**
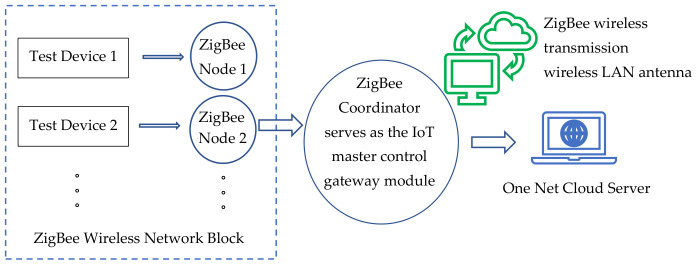
Internet of Things remote monitoring system architecture diagram.

To evaluate the performance of the designed broadband circularly polarized array antenna (referred to as “Antenna 3”) in an actual wireless communication system and compare it with two other broadband CP antennas designed concurrently in this work (“Antenna 1”: a stacked structure antenna; “Antenna 2”: a dual-band antenna), a wireless data transmission test platform based on ZigBee was established. We use the internet of things remote monitoring system architecture diagram as shown in Figure 14; the procedure is discussed as follows.

### 5.1. Test Platform and Methodology

The test platform consists of two ZigBee modules (based on the TI CC2530 chip), configured as a coordinator and an end device, respectively, operating in the 2.4 GHz band. To ensure statistical significance, the following parameters were strictly controlled:

- Test Distance: 30 m
- Transmit Power: 0 dBm
- Channel: IEEE 802.15.4 Channel 26 (2.48 GHz)
- Repetitions: 10 independent trials
- Packets per Trial: Approximately 6000 packets

On the PC, the Packet Sniffer network packet capture tool was used to count the total number of data packets sent within a fixed time and the number successfully received by the coordinator, thereby calculating the communication packet loss rate (*PLR*) by

(6) $$PLR=\frac{Ns-Nr}{Ns}\times 100\%$$

where *Ns* is number of sent packets and *Nr* is number of received packets. Testing was conducted in two distinct environments. The test platform consists of two ZigBee modules (based on the TI CC2530 chip), configured as a coordinator and an end device, respectively, operating in the 2.4 GHz band. During testing, the antenna under test (AUT) was installed on the coordinator module. The end device continuously transmits data packets, which are received by the coordinator and forwarded to a PC via an emulator. On the PC, the Packet Sniffer network packet capture tool was used to count the total number of data packets sent within a fixed time and the number successfully received by the coordinator, thereby calculating the communication packet loss rate. The *PLR* is a key metric for measuring the stability and reliability of a wireless link, calculated by the Equation (6).

Given two distinct environments, (1) Open Environment, a laboratory setting with a clear line-of-sight and minimal obstacles; (2) Complex (Multipath) Environment, a typical indoor office scene with walls, furniture, and other obstacles that induce significant multipath reflections and signal scattering, in each environment, the three designed CP antennas (Antennas 1, 2, and 3) were tested and their performance was benchmarked against a commercially available monopole linear-polarized (LP) whip antenna.

### 5.2. Test Results and Analysis

The packet loss rate test results for different antennas in the two environments are shown in Table 2.

**Open Environment:** All antennas performed excellently in the obstacle-free open environment, with extremely low packet loss rates (all below 0.35%). The performance of the three designed CP antennas was comparable to that of the whip antenna. Among them, the array antenna designed in this paper (Antenna 3) had the lowest packet loss rate (0.19%), slightly better than the others, benefiting from its higher gain and directivity.

**Complex Environment:** In the complex environment with multipath reflections and obstacles, antenna performance differed significantly. The packet loss rate of the traditional linear-polarized whip antenna sharply increased to 19.0%, severely degrading communication quality. In contrast, the three designed antennas employing CP technology demonstrated a tremendous advantage. The packet loss rates for Antenna 1, Antenna 2, and the array antenna designed in this paper (Antenna 3) were only 3.4%, 4.9%, and 3.0%, respectively, representing a reduction of approximately 15–16 percentage points compared to the whip antenna. This clearly proves the effectiveness of CP antennas in suppressing multipath interference and reducing polarization mismatch by utilizing their polarization handedness property.

**Figure 15:**
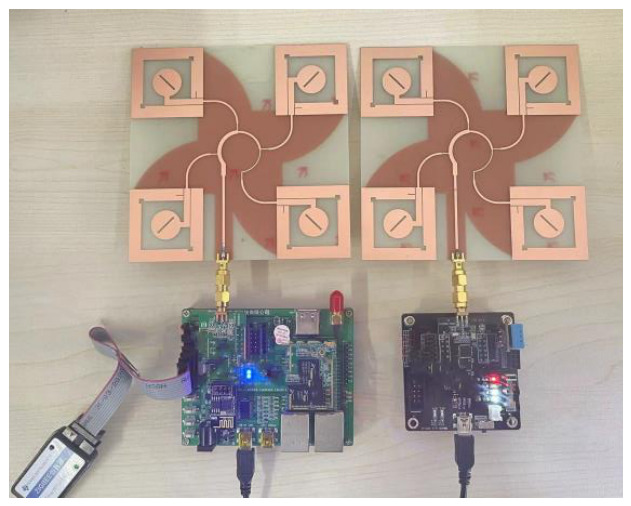
The data communication test chart of the three designed antennas.

The data communication test chart of the three designed antennas is shown in Figure 15**.** Array Antenna Advantage: In the complex environment, the designed broadband CP array antenna (Antenna 3) achieved the lowest packet loss rate (3.0%). This is not only due to its CP characteristics but also benefits from its higher gain (8.2 dBic) and stable radiation pattern, enhancing the reception capability for signals from the desired direction and further improving communication reliability under harsh channel conditions.

**Analysis:** In the complex environment, the performance spread among the three CP antennas (Antenna 1, 2, and 3) is directly correlated with their electrical characteristics. Although all utilize CP, Antenna 1 exhibits a higher PLR due to its narrower AR bandwidth (1.95–2.35 GHz), which fails to cover the full spectral width of multipath components. Antenna 2 shows the highest PLR among CP designs due to its lower gain (5.8 dBic) and reduced pattern stability. This demonstrates that CP handedness alone does not guarantee multipath immunity; gain, AR bandwidth, and pattern stability are equally critical. The proposed Antenna 3 achieves the lowest PLR due to its optimal balance of wide AR bandwidth, high gain, and stable radiation patterns.

## 6. Performance Comparison

The designed array antenna is compared with other broadband circularly polarized array antennas published in recent years, as shown in Table 3. Comparison metrics include overall size, impedance bandwidth, axial ratio bandwidth, and peak gain.

**Comparison Analysis:** It can be seen from the table that the antenna in this work holds a significant advantage in the two-core metrics of relative impedance bandwidth and relative axial ratio bandwidth. Compared to Reference [18], which operates in the same frequency band and also uses sequential rotation technology, the antenna in this work achieves a wider absolute bandwidth while maintaining comparable gain. Although References [19,20] have higher gain, they operate in the millimeter-wave band or have larger dimensions. The comprehensive performance of the antenna in this work, especially in terms of ultra-wide impedance bandwidth, validates the effectiveness of the design approach combining CPW-fed element, sequential rotation arraying, and windmill-shaped DGS in realizing a low-cost, high-performance broadband CP array antenna. Combined with the communication test results in Section 5, this work not only provides excellent parameters for the antenna itself but also verifies its tangible value in improving wireless communication quality in practical applications through system-level testing.

## 7. Conclusions

This paper successfully designed and implemented a broadband circularly polarized array antenna based on sequential rotation feeding and a windmill-shaped defected ground structure. The antenna uses an FR4 substrate, featuring a compact structure and low cost. By meticulously designing the CPW-fed circularly polarized element and utilizing sequential rotation technology for arraying, the axial ratio bandwidth was effectively broadened. The innovative introduction of the windmill-shaped DGS synergistically expanded both the impedance and axial ratio bandwidth. Measurement results show that the antenna achieves a −10 dB impedance bandwidth of 1.22–2.78 GHz (87.1% relative bandwidth) and a 3-dB axial ratio bandwidth of 1.85–2.66 GHz (35.0% relative bandwidth), with a peak gain of 8.2 dBic, the cross-polarization isolation better than 15 dB. To further evaluate its application value, the designed antenna was integrated into a ZigBee system for communication testing. The results show that in a complex multipath environment, this CP array antenna can significantly reduce the system packet loss rate from 19.0% (using a traditional linear-polarized antenna) to 3.0%, greatly enhancing the stability and reliability of the wireless link. In summary, this antenna possesses both excellent electrical performance and outstanding practical anti-interference capability, holding broad application prospects in WLAN access points, IoT gateways, and various devices requiring reliable wireless connections.

## Figures and Tables

**Table 1:** Key dimensions of the proposed antenna element (Unit: mm).

Dimension	W_0_	W_a_	W_b_	W_c_	L_d_	L_f_	L_g_
Size	40.2	14.5	3.5	4.1	1.0	2.5	22.4

**Table 2:** Packet loss rate test results for different antennas in the ZigBee communication system under open and complex multipath environments.

Antenna Type	Test Environment	Sent Packets	Received Packets	Packet Loss Rate
Whip Antenna (LP)	Open	6412	6391	0.32%
Whip Antenna (LP)	Complex	6275	5081	19.0%
Antenna 1 (CP)	Open	6331	6316	0.23%
Antenna 1 (CP)	Complex	6015	5810	3.4%
Antenna 2 (CP)	Open	5985	5969	0.26%
Antenna 2 (CP)	Complex	6112	5813	4.9%
**Antenna 3 (CP Array)**	**Open**	5641	5630	**0.19%**
**Antenna 3 (CP Array)**	**Complex**	6106	5920	**3.0%**

**Table 3:** Performance comparison of the proposed antenna with other broadband circularly polarized array antennas from recent literature.

Reference	Overall Size (mm^3^)	Impedance BW (GHz)	AR BW (GHz)	Peak Gain (dBic)
[11]	20.96 × 20.96 × 12.75	19–21 & 38–42	19–21 & 38–42	7
[12]	4.8 × 4.8 × 1.344	52.8–67.2	54.1–66.3	25.2
[13]	170 × 170 × 11.5	1.475–1.7	1.5–1.66	12.5
[14]	136 × 136 × 6	1.95–2.25	1.95–2.25	15
[15]	70 × 70 × 1.6	5.1–8.9	5.15–7.9	7.3
[16]	165.8 × 165.8 × 0.8	1.92–2.65	1.95–2.59	7.8
[17]	5.1 × 5×0.25	35.54–41.83	36–40	11.47
**This work**	**126 × 126 × 1**	**1.05–2.67**	**1.84–2.62**	**8.2**

## Data Availability

The original contributions presented in the study are included in the article, further inquiries can be directed to the corresponding author.
